# Signatures of white-matter microstructure degradation during aging and its association with cognitive status

**DOI:** 10.1038/s41598-021-83983-7

**Published:** 2021-02-25

**Authors:** Ana Coelho, Henrique M. Fernandes, Ricardo Magalhães, Pedro Silva Moreira, Paulo Marques, José M. Soares, Liliana Amorim, Carlos Portugal-Nunes, Teresa Castanho, Nadine Correia Santos, Nuno Sousa

**Affiliations:** 1 Life and Health Sciences Research Institute (ICVS), School of Medicine, University of Minho, Campus Gualtar, 4710-057 Braga, Portugal; 2 ICVS/3B’s, PT Government Associate Laboratory, 4710-057 Braga/Guimarães, Portugal; 3 Clinical Academic Center-Braga, 4710-057 Braga, Portugal; 4 Center for Music in the Brain (MIB), Aarhus University, Aarhus, Denmark; 5 Department of Psychiatry, University of Oxford, Oxford, UK

**Keywords:** Cognitive ageing, Neural ageing

## Abstract

Previous studies have shown an association between cognitive decline and white matter integrity in aging. This led to the formulation of a “disconnection hypothesis” in the aging-brain, which states that the disruption in cortical network communication may explain the cognitive decline during aging. Although some longitudinal studies have already investigated the changes occurring in white matter microstructure, most focused on specific white matter tracts. Our study aims to characterize the longitudinal whole-brain signatures of white matter microstructural change during aging. Furthermore, we assessed the relationship between distinct longitudinal alterations in white matter integrity and cognition. White matter microstructural properties were estimated from diffusion magnetic resonance imaging, and cognitive status characterized from extensive neurocognitive testing. The same individuals were evaluated at two timepoints, with a mean interval time of 52.8 months (SD = 7.24) between first and last assessment. Our results show that age is associated with a decline in cognitive performance and a degradation in white matter integrity. Additionally, significant associations were found between diffusion measures and different cognitive dimensions (memory, executive function and general cognition). Overall, these results suggest that age-related cognitive decline is related to white matter alterations, and thus give support to the “disconnected hypothesis” of the aging brain.

## Introduction

Normal aging is a heterogeneous process characterized by functional^1,2^ and structural alterations^3,4^ at the brain level, along with declines in several cognitive dimensions^5^. The “disconnection hypothesis” tries to establish a link between these age-related cognitive and brain changes, postulating that a disruption of communication between cortical regions can lead to a decline in cognitive performance^6–10^. One potential source of brain disconnection is white matter (WM) integrity and there is already evidence suggesting this relationship between WM disruption and cognitive decline in “normal” aging^11,12^.

WM integrity can be indirectly measured with diffusion tensor imaging (DTI), which measures the diffusion of water molecules in the brain^13,14^. Tissue integrity can be assessed using different DTI-based measures: fractional anisotropy (FA), mean diffusivity (MD), axial diffusivity (AD) and radial diffusivity (RD). Higher values of FA and lower values of diffusivity (MD, AD and RD) indicate higher tissue integrity^11^.

Previous cross-sectional and longitudinal studies have reported a WM degradation pattern with aging, with the most consistent findings being a decrease in FA and increase in MD with increasing age^15–21^. Other studies also report changes in RD and AD, being the observed changes usually more salient for RD than AD^22–24^ and the direction of change in AD is still controversial^25,26^. Since RD is thought to be more sensitive to myelin degeneration, it suggests that age-related WM alterations may be driven by myelination changes^11,22^. Moreover, DTI studies also report an association between WM integrity and cognitive performance within older adults, with larger effect sizes for specific cognitive dimensions, such as executive function and information processing speed (for a review see^27^). Additionally, some DTI studies report a mediation effect of WM integrity in the relationship between age and cognitive functions (e.g. associative learning, executive functions, processing speed, episodic memory)^28–33^, which suggests a causal role for WM integrity on age-related differences in cognitive performance.

Most of these studies, however, used a cross-sectional design and the existing longitudinal studies focused on specific WM tracts, despite the evidence for the existence of a global effect alongside the regional effects^11,34^. Furthermore, a previous study from our lab^12^ used DTI to assess which WM microstructural properties could discriminate between different profiles of cognitive performance, in a cross-sectional design. In this study, we continue this previous work, using longitudinal data from the same group of individuals. Our main goal was to investigate: (1) WM integrity changes along time and (2) associations between WM microstructure alterations and cognition. We hypothesized that aging would trigger a degradation of white-matter integrity and this deterioration will be associated to cognitive decline. To test this, we estimated white-matter microstructural properties of a group of older adults that were followed longitudinally from diffusion MRI. We characterized cognitive status from extensive neurocognitive testing and tested relationships between longitudinal changes in white-matter integrity and cognition.

## Methods

### Ethics statement

The present study was conducted in accordance with the principles expressed in the Declaration of Helsinki and was approved by the national ethical committee (Comissão Nacional de Proteção de Dados) and by the local ethics review boards (Hospital de Braga, Braga; Centro Hospitalar do Alto Ave, Guimarães; and Unidade Local de Saúde do Alto Minho, Viana-do-Castelo/Ponte-de-Lima). The study goals and procedures were explained to the participants and all gave informed written consent.

### Participants

The participants included in this study are part of the sample recruited for the SWITCHBOX Consortium project (www.switchbox-online.eu/). These participants were recruited from a larger sample randomly selected from Guimarães and Vizela local area health authority registries, that is representative of the general Portuguese population for age, gender and education^35–37^. Primary exclusion criteria were inability to understand the informed consent, participant’s choice to withdraw from the study, incapacity and/or inability to attend the MRI session, dementia and/or diagnosed neuropsychiatric and/or neurodegenerative disorder and/or cerebrovascular disease (medical records). Mini Mental State Examination (MMSE) scores below the adjusted thresholds for cognitive impairment were also used as exclusion criteria. Following recommendations, the thresholds were adjusted depending on factors such as age and/or education^38,39^. This resulted in the following adjusted thresholds for cognitive impairment: MMSE score < 17 if individual with ≤ 4 years of formal school education and/or ≥ 72 years of age, and MMSE score < 23 otherwise (follows the MMSE validation study for the Portuguese population)^40^.

In the first assessment, 100 subjects were contacted for MRI screening. From these, three subjects did not finish the diffusion acquisition and four subjects were excluded after the visual inspection of the MRI scans by a certified neuroradiologist concluded that they had brain lesions/pathology. For the last assessment, all participants from the baseline were contacted to perform the follow-up evaluation but subjects presenting diseases that could affect both cognition and white matter microstructure (e.g., cerebrovascular disease) were excluded. In the end, 55 subjects accepted to be re-evaluated and were able to perform the MRI acquisition protocol, but one did not finish the diffusion acquisition. A total of 51 individuals with diffusion data from both the first and last assessments met all the inclusion criteria for this study.

### Neurocognitive assessment

A team of certified psychologists performed a battery of neurocognitive tests in the two timepoints. This included the following tests validated for the Portuguese population: Stroop color and word test, selective reminding test (SRT) and mini-mental state examination (MMSE). Stroop test was used to evaluate cognitive flexibility and inhibitory control and it was composed of three parameters: words (SW), colors (SC) and words/colors (SWC) evaluated at three parts. In the first part, different words of colors printed in black are presented to the participant which has to read the word (Stroop words). In the second part, the ‘XXXX’ word is printed in different ink colors and the participant has to name the ink color (Stroop colors). The third and final part corresponds to the interference (Stroop words/colors) component of the test, where the participant is presented with inconsistent association between the word and the ink in which the word is printed (e.g., word “blue” printed in red ink). In this case, the participant has to name the ink color instead of reading the word. SRT was also constituted of three variables: long-term storage (LTS), consistent long-term retrieval (CLTR) and delayed-recall (DR) and assessed verbal learning and memory. MMSE was performed to evaluate general cognition through the assessment of different cognitive domains, such as orientation, word recall, attention and calculation, language and visual-construction abilities. Test scores were transformed to be expressed in the same scale. Since we are dealing with longitudinal data, z-score standardization has some limitations, namely the loss of information about mean-level changes across time. In order to overcome this issue, we transformed test scores using the proportion of maximum scaling (POMS), according to the formula:

$$POMS = \frac{observed - minimum}{{maximum - minimum}}$$

The transformed test scores range from 0 (minimum possible value) to 1 (maximum possible value)^41^.

### MRI data acquisition

All MRI assessments were performed at Hospital de Braga (Braga, Portugal) on a clinical approved Siemens Magnetom Avanto 1.5 T MRI scanner (Siemens Medical Solutions, Erlangen, Germany) with a 12-channel receive-only head-coil. The imaging protocol included several different acquisitions. For the present study, only the Diffusion Weighted Imaging (DWI) acquisition was considered. For this, a spin-echo echo-planar imaging (SE-EPI) sequence was acquired with the following parameters: TR = 8800 ms, TE = 99 ms, FoV = 240 × 240 mm, acquisition matrix = 120 × 120, 61 2-mm axial slices with no gap, 30 non-collinear gradient direction with b = 1000 s mm^−2^, one b = 0 s mm^−2^ and 1 repetition.

All acquisitions were visually inspected by a certified neuroradiologist, before data pre-processing, to ensure that none of the individuals included in this study had brain lesions and/or critical head motion or artifacts that could comprise the quality of the data and reliability of our findings.

### DWI data pre-processing and tensor fitting

All data was pre-processed using FMRIB Diffusion Toolbox (FDT) provided with the FMRIB Software Library (FSL v5.0; https://fsl.fmrib.ox.ac.uk/fsl/). Pre-processing included: correction for motion and eddy current distortions; rotation of gradient vectors accordingly to the affine transformations used to register each volume; extraction and skull stripping of the first b0 volume of each subject; removal of non-brain structures by applying the brain mask created in the previous step to the remaining volumes.

Tensor fitting and scalar maps computation steps were performed with DTIFIT that is part of FDT toolbox. Briefly, DTIFIT fits a diffusion tensor model at each voxel and generates scalar maps of FA and MD, as well as eigenvector and eigenvalues maps. AD scalar map was defined as the principal diffusion eigenvalue and RD as the mean of the second and third eigenvalues.

### Longitudinal tract-based spatial statistics

Voxel-wise analysis of scalar maps across subjects and timepoints was performed using TBSS procedures^42^, part of FSL. While this method aims to solve issues of aligning data from multiple subjects, it does not take into account variation from multiple timepoints. Thus, in this study, we implemented a modified TBSS pipeline that improves anatomical longitudinal alignment, as described in^43^. First, linear transformations between the b0 images of the first and second timepoints were computed using FLIRT. Then, the b0 images and scalar maps of both timepoints were resampled to a space halfway between the two, that was previously computed with MIDTRANS. Next, a subject-wise mid-space template was created by averaging the two halfway registered FA-maps. These subject’s templates were then used in the normal TBSS procedures. Initially, each subject’s FA template was slightly eroded, and the end slices were zeroed in order to further remove potential outliers. Next, all FA templates were nonlinearly registered into a 1 × 1 × 1 mm standard space. In order to accomplish this particular step, each subject’s FA template was nonlinear registered to each other to find the “most representative one” (i.e., the one that requires the least warping to align all images) that served as the study specific target image. Then, the chosen target was affine transformed into Montreal Neurological Institute (MNI) 152 standard space and each subject’s FA template was transformed into standard space through the combination of the nonlinear transformation to the study specific target with the affine transformation into MNI space. Next, FA templates of all subjects were averaged, and the resulting image skeletonized and thresholded. Thresholding the mean FA value between 0.2 and 0.3 was found to successfully remove from the skeleton regions encompassing multiple tissue types^42^. Thus, after visual inspection we thresholded the skeleton image at 0.3. Finally, all scalar maps (FA, AD, MD and RD) from the two time points were projected into this FA skeleton using the same transformation applied to the FA templates.

### Statistical analysis

Statistical analysis of the skeletonized maps of FA, AD, MD and RD was performed in order to discriminate which WM tracts exhibit statistically significant differences. This was accomplished using the permutation methods employed in “randomise”, distributed with FSL. We performed a paired sample t-test to investigate age-related trajectories of WM microstructure. Five thousand random permutations were used in the inference of the contrasts of interest. Widespread significant differences were detected with threshold-free cluster enhancement (TFCE), whereas multiple comparisons were corrected using family-wise error rate (FWE-R) at α = 0.05. Clusters showing significant results were labeled according to the Johns Hopkins University ICBM-DTI-81 WM labels atlas^44^ and dilated with tbss_fill tool (distributed with FSL) for visualization purposes. We also calculated the Dice coefficient between each pair of significant clusters to evaluate the degree of similarity between them. Dice coefficient ranges between 0 and 1, with values of 1 meaning that the two clusters are a perfect match. Subsequently, we investigated the associations between the significant results from this analysis and the scores of neurocognitive tests. To do this, the mean DTI metrics (FA, AD, MD and RD) were extracted from each significant cluster of each scontrast of interest and correlation analyses were performed between the mean DTI metric and each cognitive test, for all timepoints. Subjects with missing values in cognitive scores were excluded from the analyses and p-values were corrected for multiple comparisons, using the false discovery rate (FDR) method. The rmcorr R package (https://cran.r-project.org/web/packages/rmcorr/) was used to compute a repeated measures correlation coefficient between each DTI metric and cognitive score. Repeated measures correlation analysis computes the correlation within each individual between two variables measured longitudinally and then estimates the common regression slope, which is the association shared between individuals. This technique takes into account non-independence between observations of repeated measures data and has greater statistical power than the standard Pearson correlation coefficient using averaged data^45^.

Additionally, we calculated the percentage of change between timepoints for each significant cluster of each DTI metric, and for each cognitive test score. The following formula was used:

$$\Delta_{ij} = \frac{{M_{j} - M_{i} }}{{\left| {M_{i} } \right|}} \times 100$$

where $M_{i}$ and $M_{j}$ are the metric values at timepoint $i$ and $j$, respectively. We also tested differences between slopes of each significant cluster. For this, we first performed a linear regression for each cluster and then an Analysis of Covariance (ANCOVA) with DTI metric as dependent variable, cluster as the factor and timepoint as the covariate and analyzed the significance of the interaction term. We transformed values to reflect only increases in time, in order to compare slopes independently of the direction.

### Ethical approval

All procedures followed were in accordance with the ethical standards of the responsible committee on human experimentation (institutional and national) and with the Helsinki Declaration of 1975, and the applicable revisions at the time of the investigation. Informed consent was obtained from all patients for being included in the study.

## Results

### Sample characteristics

Table 1 shows the demographic characterization of the participants included in this study. In summary, mean age at baseline was 63.5 years (range 51–82 years) and mean interval between evaluations was 52.8 months (range 45–73 months). Interval time was not significantly associated with age at baseline (r = − 0.12, p = 0.41). The sample was balanced for males and females (51% females, 49% males) and they did not differ with respect to interval time ($t\left( {30} \right) = 0.14, p = 0.89$). Mean education level was 5.98 years (range 0–17 years). Regarding neurocognitive test scores, all variables show a decrease along time, with the exception of Stroop words parameter that remains constant (Supplementary Fig. 1). Statistically significant differences were found between timepoints for long-term storage ($t\left( {50} \right) = 3.40, p = 0.003, d = 0.48$), Stroop colors ($t\left( {50} \right) = 4.48, p = 0.0003, d = 0.63$) and MMSE variables ($t\left( {50} \right) = 4.04, p = 0.0006, d = 0.57$).

**Table 1:** Basic demographic and cognitive characterization of the study’s cohort.

	Baseline Mean ± SD (range)	Follow-up Mean ± SD (range)	Test statistic
N (females/males)	51 (26/25)	–	–
Age (years)	63.5 ± 7.41 (51–82)	68.0 ± 7.25 (55–86)	
Interval (months)	52.8 ± 7.24 (45–73)	–	
Education (years)	5.98 ± 3.97 (0–17)	–	
LTS	0.53 ± 0.24 (0.069–1)	0.43 ± 0.24 (0–1)	t(50) = 3.40, p = 0.003**, d = 0.48
CLTR	0.38 ± 0.26 (0–1)	0.33 ± 0.25 (0–1)	t(50) = 1.86, p = 0.096
DR	0.50 ± 0.27 (0–1)	0.45 ± 0.25 (0–1)	t(50) = 1.11, p = 0.32
SW	0.55 ± 0.27 (0.013–1)	0.55 ± 0.22 (0–1)	t(50) = 0.30, p = 0.77
SC	0.63 ± 0.21 (0–0.99)	0.53 ± 0.26 (0–1)	t(50) = 4.48, p = 0.0003***, d = 0.63
SWC	0.53 ± 0.23 (0–1)	0.49 ± 0.24 (0–1)	t(50) = 1.98, p = 0.094
MMSE	0.82 ± 0.22 (0–1)	0.72 ± 0.23 (0.077–1)	t(50) = 4.04, p = 0.0006***, d = 0.57

Statistical results FDR corrected at p < 0.05.

*LTS* long-term storage, *CLTR* consistent long-term retrieval, *DR* delayed-recall, *SW* Stroop words, *SC* Stroop colors, *SWC* Stroop words/colors, *MMSE* Mini-Mental State Examination.

Significance codes: *p < 0.05, **p < 0.01, ***p < 0.001.

### Age-related trajectories in WM microstructure

**Figure 1:**
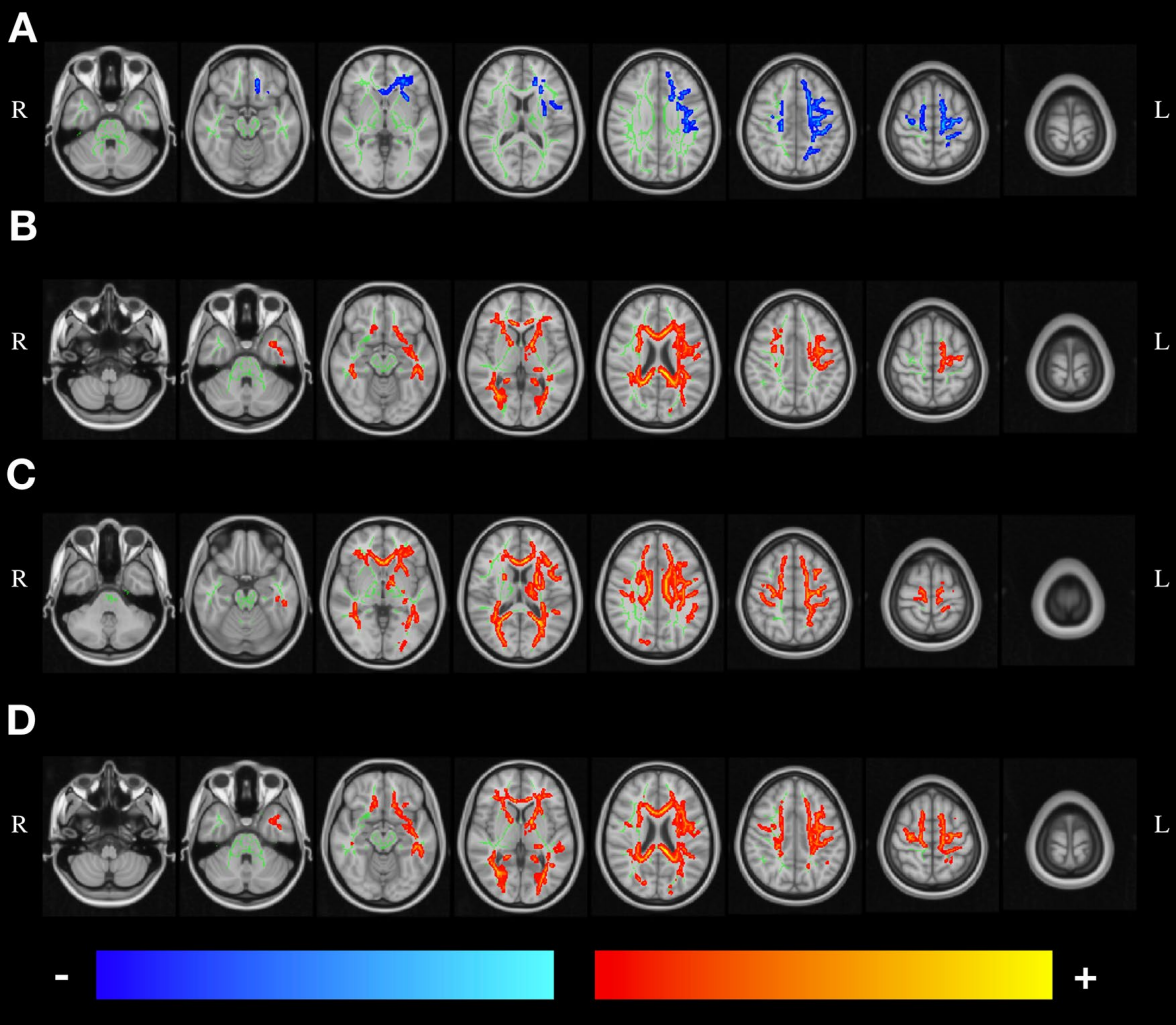
Statistically significant changes along time in (**A**) FA, (**B**) AD, (**C**) RD and (**D**) MD maps. Blue/light-blue gradient indicates decreases along time. Red/yellow gradient indicates increases along time. All results were considered significant at p < 0.05 (FWE corrected for multiple comparisons). We observe a decrease in FA with a left hemisphere dominant pattern, while the other metrics (AD, RD and MD) exhibit an increase between timepoints with the changes being spread throughout the brain.

Statistical analysis of the skeletonized maps revealed statistically significant differences between timepoints for all DTI metrics (Fig. 1). Significant longitudinal decreases for the FA maps were found in two clusters. One of these clusters (Cluster 1) comprises the body of corpus callosum, and right superior and posterior corona radiata (Supplementary Fig. 2A). The other cluster (Cluster 2) includes the genu, body and splenium of corpus callosum, anterior limb of internal capsule, anterior, superior and posterior corona radiata, external capsule and superior longitudinal fasciculus, with most of these tracts located in the left hemisphere (Supplementary Fig. 2B). For AD and MD metrics, significant longitudinal increases were found in a large cluster spread throughout the brain. This cluster includes WM tracts such as the genu, body and splenium of corpus callosum, cerebral peduncle, internal capsule, corona radiata, posterior thalamic radiation, sagittal stratum, external capsule, cingulum, fornix, superior longitudinal fasciculus, superior fronto-occipital fasciculus, tapetum and uncinate fasciculus. Regarding RD, two clusters were found, with one of them comprising only the right superior longitudinal fasciculus (Cluster 1) (Supplementary Fig. 2C), while the other (Cluster 2) includes the same tracts as the cluster found for AD and MD (Supplementary Fig. 2D). A summary of these results, with cluster size, coordinates and corresponding white matter tract of the peak are present in Supplementary Table 1.

Regarding the similarity of the obtained significant clusters, measured with Dice coefficient, we found that FA Cluster 2 and RD Cluster 1 did not overlap with any other cluster. FA Cluster 1 had a small overlap with AD, MD and RD Cluster 2, while FA Cluster 2 had increased similarity with the same clusters. AD, MD and RD Cluster 2 had the highest degrees of similarity between them. A summary of these results is present in Table 2.

**Table 2:** Dice coefficient between each pair of significant clusters.

Metric	FA cluster 1	FA cluster 2	AD cluster 1	MD cluster 1	RD cluster 1	RD cluster 2
FA cluster 1	1	–	–	–	–	–
FA cluster 2	0	1	–	–	–	–
AD cluster 1	0.001	0.22	1	–	–	–
MD cluster 1	0.060	0.37	0.76	1	–	–
RD cluster 1	0	0	0	0	1	–
RD cluster 2	0.072	0.42	0.63	0.87	0	1

**Figure 2:**
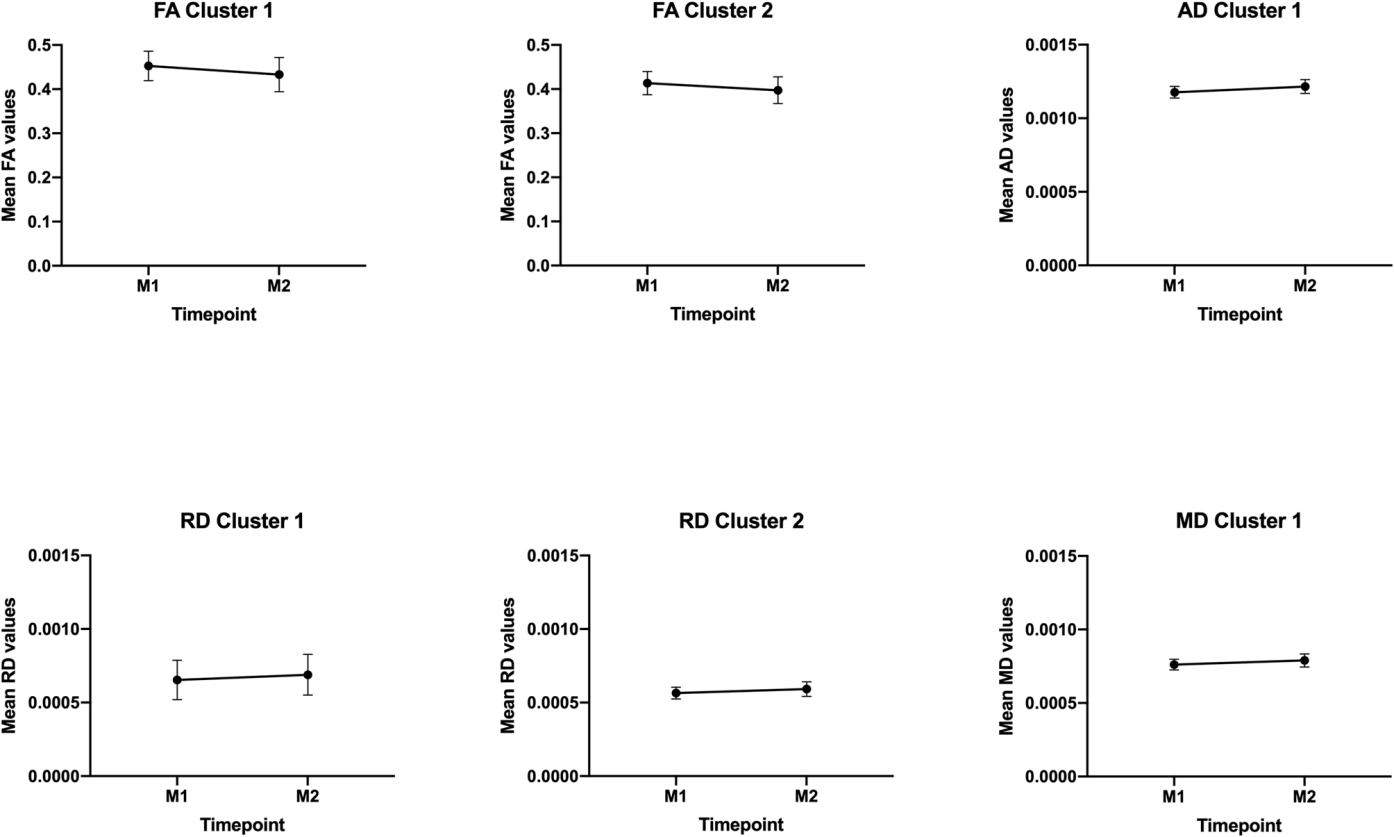
Trajectories of DTI metrics (FA, AD, RD and MD) of each cluster with significant differences between timepoints. The x-axis represents time of assessment and y-axis, the average values of each metric. Error bars represent standard deviation. FA clusters show a decrease along time, while AD, RD and MD clusters exhibit an increase. Overall, these results suggest that aging induces a deterioration of white matter integrity.

Our analysis of the mean DTI metrics of the significant clusters revealed a linear decrease of FA and a linear increase of AD, MD and RD, from the first to last timepoint (Fig. 2). All slopes were significantly different from zero, with the exception of the first cluster of RD. Table 3 presents the percentages of change between timepoints, the linear regression slopes and the significance of each slope for the different DTI metrics of these significant clusters. We found a significant interaction in the relationship of DTI metric to time for the different clusters ($F\left( {5,600} \right) = 6.35, p < 0.001$), which suggests that there are differences in the slopes. Post-hoc tests with Bonferroni correction revealed that slopes of both FA clusters were significantly different from slopes of the other metrics (AD, RD and MD) (Supplementary Table 2). We can see that slopes of FA clusters are three orders of magnitude higher in comparison to the other metrics, but in terms of percent of change between timepoints, the values are similar for all metrics.

**Table 3:** Percentage of longitudinal changes in DTI metrics, linear regression slopes and significance of slopes of each significant cluster.

Metric	M2–M1 (%)	Slope	Test statistic
FA cluster 1	− 4.35	− 0.020	F(1,100) = 7.62, p = 0.007**
FA cluster 2	− 3.94	− 0.016	F(1,100) = 8.42, p = 0.005**
AD cluster 1	3.30	0.00004	F(1,100) = 20.51, p < 0.0001***
RD cluster 1	5.45	0.00004	F(1,100) = 1.74, p = 0.19
RD cluster 2	4.78	0.00003	F(1,100) = 9.09, p = 0.003**
MD cluster 1	3.69	0.00003	F(1,100) = 11.96, p = 0.0008***

*FA* fractional anisotropy, *AD* axial diffusivity, *MD* mean diffusivity, *RD* radial diffusivity, *M1* timepoint 1, *M2* timepoint 2.

Significance codes: *p < 0.05, **p < 0.01, ***p < 0.001.

### Associations with cognition

**Figure 3:**
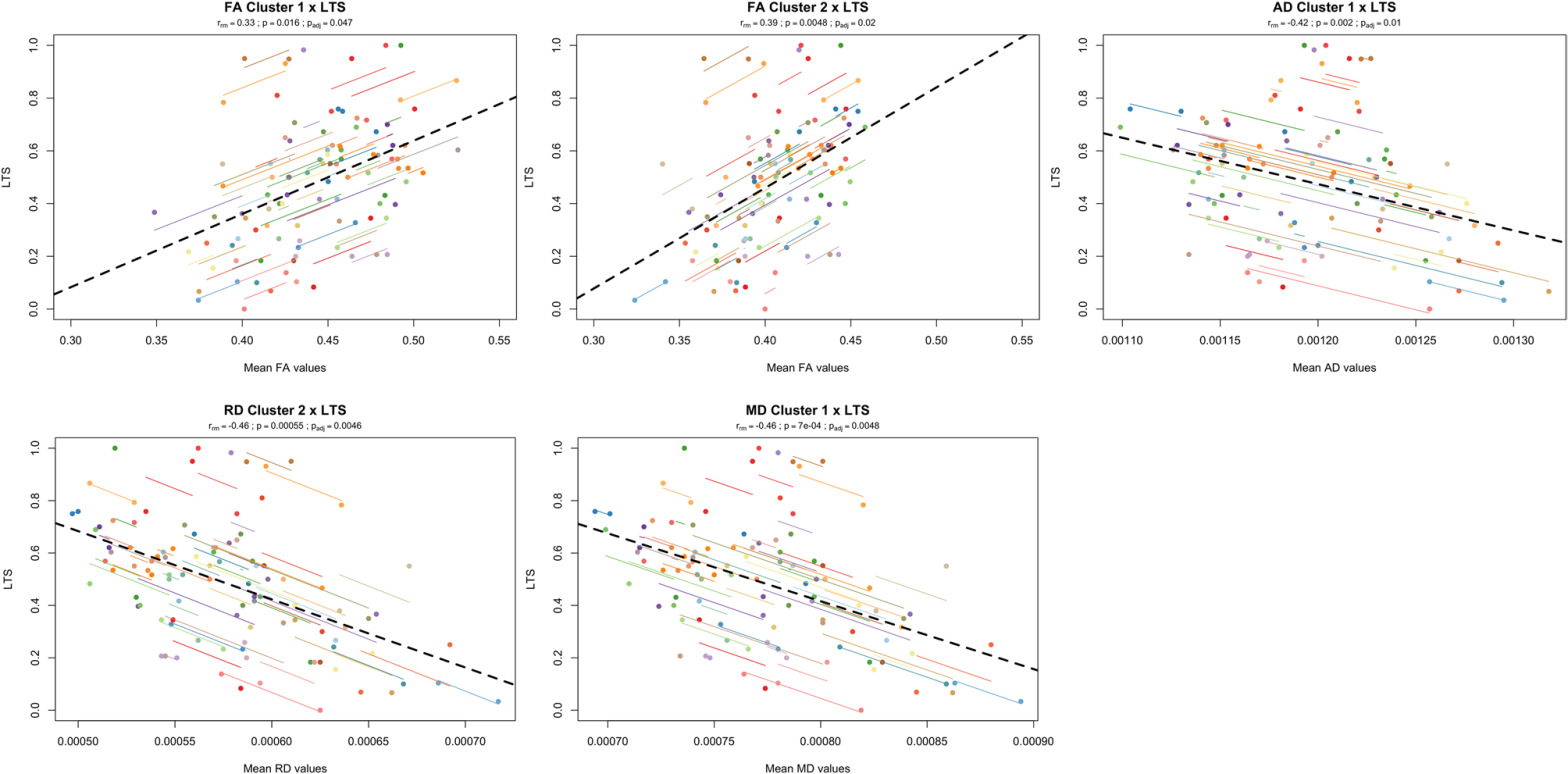
Significant repeated measures correlations between long-term storage (LTS) test score and DTI metrics (FA, AD, RD and MD) of clusters with significant changes between timepoints. The x-axis represents average values of each DTI metric and y-axis, the average values of LTS. Observations from the same individual are represented with the same color, with corresponding lines showing the repeated measures correlation fit for each subject. Dashed black line represents the overall regression line. All clusters, with the exception of RD cluster 1, were significantly associated with LTS. For FA clusters, we found a positive correlation, meaning that higher FA values are associated with higher LTS scores. For AD, RD and MD clusters, a negative correlation was found, showing that lower AD, RD or MD values are associated with higher LTS scores. Overall, these results suggest that higher WM integrity is associated with higher cognitive performance in the memory domain.

**Figure 4:**
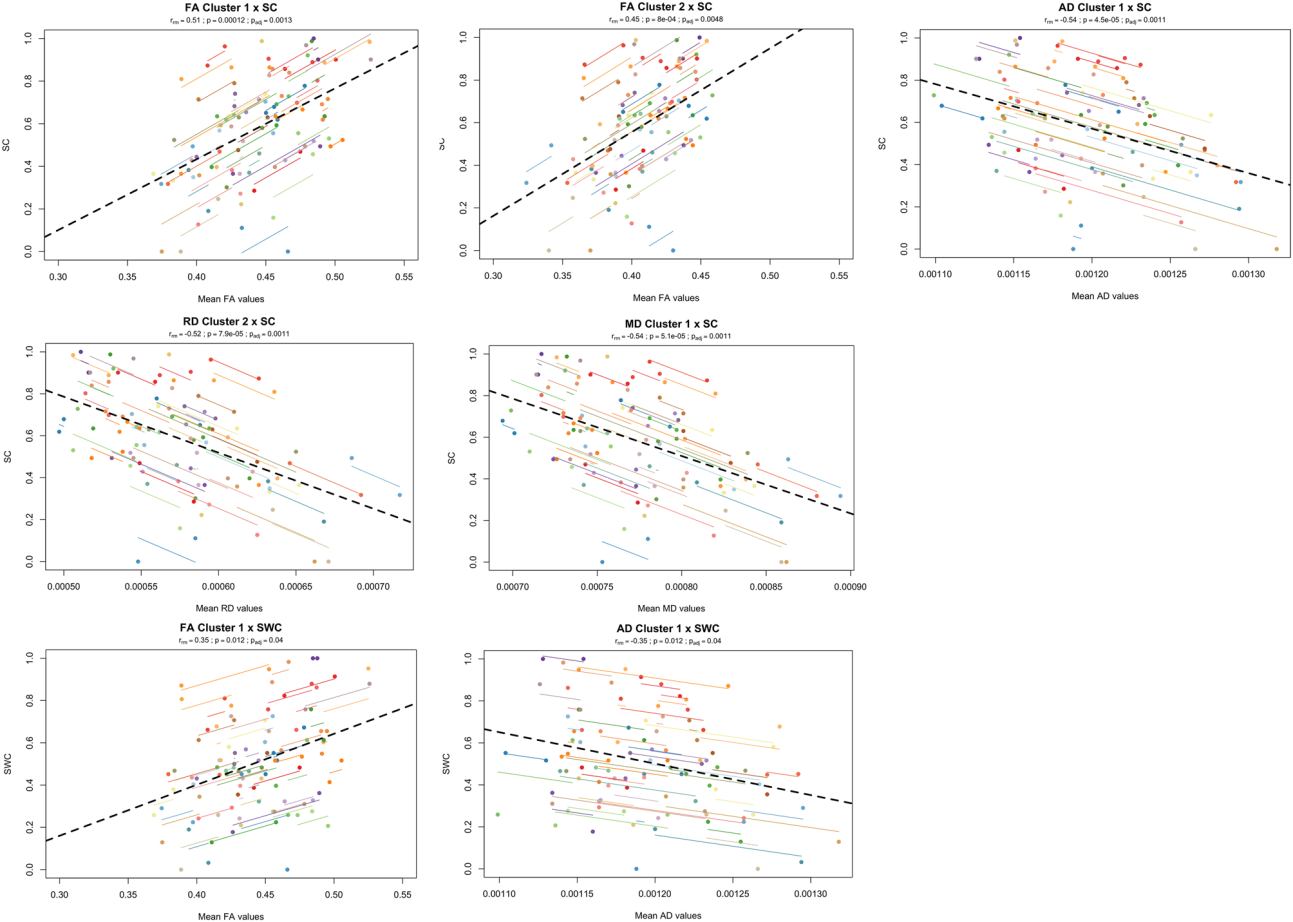
Significant repeated measures correlations between Stroop test variables (Stroop colors – SC, Stroop words/colors – SWC) and DTI metrics (FA, AD, RD and MD) of clusters with significant changes between timepoints. The x-axis represents average values of each DTI metric and y-axis, the average values of SC/SWC. Observations from the same individual are represented with the same color, with corresponding lines showing the repeated measures correlation fit for each subject. Dashed black line represents the overall regression line. All clusters, with the exception of RD cluster 1, were significantly associated with SC. For FA clusters, we found a positive correlation, meaning that higher FA values are associated with higher LTS scores. For AD, RD and MD clusters, a negative correlation was found, showing that lower AD, RD or MD values are associated with higher SC scores. Regarding SWC, FA cluster 1 was positively correlated and AD was negatively correlated. Overall, these results suggest that higher WM integrity is associated with higher cognitive performance in the executive function domain.

**Figure 5:**
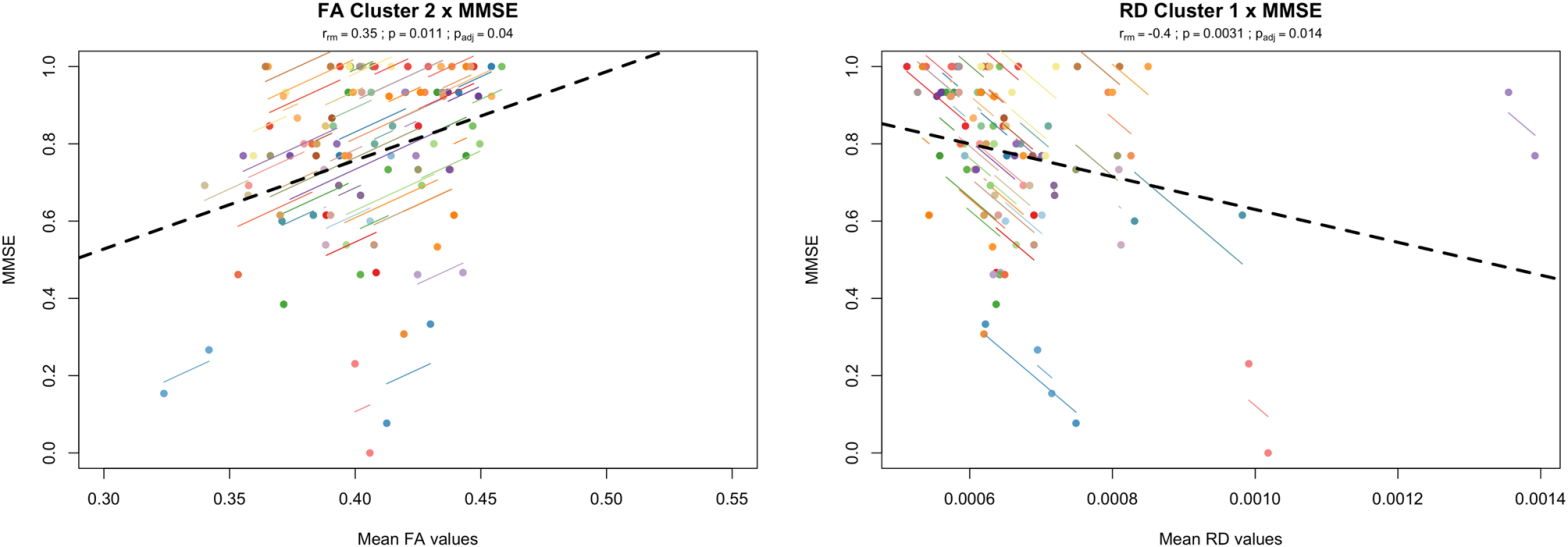
Significant repeated measures correlations between Mini-Mental State Examination (MMSE) and DTI metrics (FA and RD) of clusters with significant changes between timepoints. The x-axis represents average values of each DTI metric and y-axis, the average values of MMSE. Observations from the same individual are represented with the same color, with corresponding lines showing the repeated measures correlation fit for each subject. Dashed black line represents the overall regression line. Only FA cluster 2 and RD cluster 1 were significantly associated with MMSE. FA exhibited a positive correlation, meaning that higher FA values are associated with higher MMSE scores. RD was negatively correlated with MMSE, showing that lower RD values are associated with higher MMSE scores. Overall, these results suggest that higher WM integrity is associated with higher general cognition.

Significant correlations were found for the different DTI metrics and some of the neurocognitive variables. Specifically, LTS parameter was significantly associated with both FA clusters (FA Cluster 1—r = 0.33, p = 0.047; FA Cluster 2—r = 0.39, p = 0.020), AD cluster (r = -0.42, p = 0.010), RD cluster 2 (r = − 0.46, p = 0.005) and MD cluster (r = − 0.46, p = 0.005) (Fig. 3). The other parameters of SRT (CLTR and DR) did not display any significant correlation with DTI metrics. Regarding Stroop test, SC was significantly correlated with both FA clusters (FA Cluster 1—r = 0.51, p = 0.001; FA Cluster 2—r = 0.45, p = 0.005), AD cluster (r = − 0.54, p = 0.001), RD cluster 2 (r = − 52, p = 0.001) and MD cluster (r = − 54, p = 0.001), while SWC had significant correlations with FA cluster 1 (r = 0.35, p = 0.04) and the AD cluster (r = − 0.35, p = 0.04) (Fig. 4). Finally, MMSE was significantly associated with FA cluster 2 (r = 0.35, p = 0.04) and RD cluster 1 (r = − 0.40, p = 0.01) (Fig. 5). Table 4 summarizes results of all correlations performed. Interestingly, the cognitive variables with significant associations have the higher rates of decrease along time, with the exception of CLTR that has the third highest rate of decrease but no significant correlation with any DTI metric (Table 5).

**Table 4:** Correlations between DTI metrics and scores of cognitive tests at all timepoints (results FDR corrected at p < 0.05).

	FA cluster 1	FA cluster 2	AD cluster 1	RD cluster 1	RD cluster 2	MD cluster 1
LTS	r = 0.33* (p = 0.047)	r = 0.39* (p = 0.020)	r = − 0.42* (p = 0.010)	r = **− **0.31 (p = 0.069)	r = − 0.46** (p = 0.005)	r = − 0.46** (p = 0.005)
CLTR	r = 0.17 (p = 0.32)	r = 0.19 (p = 0.28)	r = **− **0.25 (p = 0.13)	r = **− **0.13 (p = 0.44)	r = **− **0.25 (p = 0.13)	r = **− **0.25 (p = 0.13)
DR	r = 0.079 (p = 0.66)	r = 0.14 (p = 0.40)	r = **− **0.072 (p = 0.68)	r = **− **0.16 (p = 0.35)	r = **− **0.13 (p = 0.44)	r = **− **0.10 (p = 0.55)
SW	r = 0.15 (p = 0.37)	r = 0.017 (p = 0.91)	r = 0.057 (p = 0.72)	r = **− **0.16 (p = 0.36)	r = 0.053 (p = 0.73)	r = 0.063 (p = 0.71)
SC	r = 0.51** (p = 0.001)	r = 0.45** (p = 0.005)	r = − 0.54** (p = 0.001)	r = **− **0.33 (p = 0.054)	r = − 0.52** (p = 0.001)	r = − 0.54** (p = 0.001)
SWC	r = 0.35* (p = 0.040)	r = 0.28 (p = 0.092)	r = − 0.35* (p = 0.040)	r = **− **0.21 (p = 0.23)	r = **− **0.30 (p = 0.083)	r = **− **0.31 (p = 0.066)
MMSE	r = 0.26 (p = 0.12)	r = 0.35* (p = 0.040)	r = **− **0.20 (p = 0.26)	r = − 0.40* (p = 0.014)	r = **− **0.28 (p = 0.092)	r = **− **0.25 (p = 0.13)

*FA* fractional anisotropy, *AD* axial diffusivity, *MD* mean diffusivity, *RD* radial diffusivity, *LTS* long-term storage, *CLTR* consistent long-term retrieval, *DR* delayed-recall, *SW* Stroop words, *SC* Stroop colors, *SWC* Stroop words/colors, *MMSE* Mini-Mental State Examination.

Significance codes: *p < 0.05, **p < 0.01, ***p < 0.001.

**Table 5:** Percentage of longitudinal changes in neurocognitive test scores.

Metric	M2–M1 (%)
LTS	− 17.8
CLTR	− 14.6
DR	− 9.48
SW	− 0.27
SC	− 16.5
SWC	− 7.40
MMSE	− 11.9

*LTS* long-term storage, *CLTR* consistent long-term retrieval, *DR* delayed-recall, *SW* Stroop words, *SC* Stroop colors, *SWC* Stroop words/colors, *MMSE* Mini-Mental State Examination, *M1* timepoint 1, *M2* timepoint 2.

## Discussion

Herein we explore the effect of age on WM microstructure, by combining diffusion magnetic resonance imaging with neurocognitive testing. Our results reveal that aging is characterized by a degradation in white matter integrity and cognitive decline. Furthermore, we found significant associations between diffusion measures and cognitive dimensions of memory, executive function and general cognition. In sum, these findings are in line with the “disconnection” hypothesis of the aging brain, by demonstrating a relationship between white matter integrity deterioration and cognitive decline.

Our analysis of the DTI metrics revealed decreased FA and increased diffusivity (AD, MD and RD) with aging in brain areas where these parameters are statistically different across the 52.8 months of observation, which is consistent with previous longitudinal studies^15,16^. While for diffusivity measures, the results were relatively widespread, comprising several WM tracts in both hemispheres, for FA the results were localized. Decreased FA with aging was found in corpus callosum, left anterior limb of internal capsule, left external capsule, left/right corona radiata and left superior longitudinal fasciculus, which have already been reported in previous longitudinal studies^2,15^. This pattern of left-dominant deterioration was already reported in studies with cognitively impaired patients. Specifically, a rightward lateralization of functional connectivity in patients with mild cognitive impairment (MCI) and Alzheimer’s Disease (AD) has been reported^46^, which is possibly due to a compensation mechanism for the loss of cognitive function or because of disease-induced damage in the left hemisphere. Furthermore, Low and colleagues^47^ showed the existence of a higher degree of asymmetry of white matter hyperintensities towards the left hemisphere in AD patients, when compared to MCI and healthy controls, which is associated with poorer global cognition, memory, executive function and language.

Correlation analysis between cognitive scores and age-related WM changes showed significant associations between all diffusion measures (FA, AD, RD and MD), and cognitive variables of memory (LTS) and executive function (SWC). These results may suggest that longitudinal changes in cognition are associated with changes in WM integrity, thus supporting the “disconnection hypothesis”. This association has already been extensively reported in the literature, with several studies showing the existence of a clear association between WM integrity deterioration and poorer performance in executive function tasks^29,48–53^, with results spanning over different regions in the brain. Particularly, Hedden and colleagues^50^ found a mediation effect of WM integrity, along with cortical thickness and glucose metabolism, in age-related differences in executive function. Noticeably, the majority of the studies report larger effect sizes for associations between WM integrity and cognitive dimensions, such as executive function and processing speed, than for memory^11,27^. Still, there is evidence of a relation between WM integrity and memory^22,31,48,51,52,54^. In our study, the effect sizes for memory and executive function are very similar. We also revealed significant associations between diffusion measures and the color naming parameter (SC) of the Stroop test. Although only the interference parameter (SWC) measures executive function, the other two parameters (SW and SC) are considered measures of processing speed^55^. Previous studies found significant age effects in the SC parameter, which can be the result of a general slowing induced by age^56^. Uttl and colleagues also found that the age effects on the interference condition was directly related to the performance on the other two conditions. Thus, the longitudinal changes that we observe in the interference component might be attributed to longitudinal changes in the performance of the color naming task. Regarding general cognition, evaluated through MMSE, significant correlations were found for FA and RD. This result is in accordance with previous literature showing that age effects on WM integrity are often stronger for RD than AD^22–24^. Davis and colleagues^22^, in addition to this, also found that the impact of age-related WM changes in cognitive performance was more prominent for RD than for AD. Additionally, the cognitive variables revealing significant associations with WM integrity presented accentuated declines in subsequent observations, which indicates that steeper declines in cognition are associated with steeper declines in WM integrity along time. Once again, this reinforces the theory that deterioration of WM causes a disruption in the communication between cortical regions, which, in turn, leads to cognitive decline.

One of the limitations of this study was the use of a whole-brain approach to investigate alterations in WM integrity. Although this allowed us to explore the global effect of aging in WM microstructure and its relationship with cognition, it was not possible to examine the contribution of each individual WM tract. Future work may include the analysis of age effects in the integrity of each WM tract and its association with cognitive function. Another limitation is the use of a 1.5 T MRI scanner which has lower signal to noise ratio (SNR) when compared to 3 T MRI scanners^57^. Furthermore, the parameters used to acquire the DWI sequence, namely 30 gradients directions and only a single b = 0 s mm^−2^ volume, also impact on the resolution diffusion data.

In summary, our findings confirm the existing evidence of a degradation of the WM with aging. We also found significant associations between DTI metrics and the different cognitive dimensions evaluated (memory, executive function and general cognition). This result indicates a relationship between age-related changes in WM microstructural properties and cognitive function, which brings further support to the “disconnection hypothesis”. Furthermore, this association between the effects of aging on WM integrity and cognition is only possible with the use of a longitudinal design^1^, such as what we present here. Finally, our findings open new perspectives for future studies to identify the main drivers in WM integrity levels at different levels of cognitive ability. Hence, this could help in the development of new in-vivo brain biomarkers of inter-individual variability in cognitive trajectories.

## Supplementary Information

Supplementary Information
