# Supplementary material for: Signatures of white-matter microstructure degradation during aging and its association with cognitive status
Source: Sci Rep. 2021 Feb 25;11:4517. doi: 10.1038/s41598-021-83983-7 (PMC7907273; doi:10.1038/s41598-021-83983-7)
Supplement: Supplementary file 1 — Supplementary Information [file 41598_2021_83983_MOESM1_ESM.docx]

**Signatures of White-matter Microstructure Degradation During Aging and its Association with Cognitive Status**

Ana Coelho^1,2,3^, Henrique M. Fernandes^4,5^, Ricardo Magalhães^1,2,3^, Pedro Silva Moreira^1,2,3^, Paulo Marques^1,2,3^, José M. Soares^1,2,3^, Liliana Amorim^1,2,3^, Carlos Portugal-Nunes^1,2,3^, Teresa Castanho^1,2,3^, Nadine Correia Santos^1,2,3^, and Nuno Sousa^1,2,3^

^1^Life and Health Sciences Research Institute (ICVS), School of Medicine, University of Minho, 4710-057 Braga, Portugal.

^2^ICVS/3B’s, PT Government Associate Laboratory, 4710-057 Braga/Guimarães, Portugal.

^3^Clinical Academic Center – Braga, 4710-057 Braga, Portugal.

^4^Center for Music in the Brain (MIB), Aarhus University, Aarhus, Denmark

^5^Department of Psychiatry, University of Oxford, Oxford, UK

*** Correspondence:**

Nuno Sousa, MD/PhD, Life and Health Sciences Research Institute (ICVS), School of Medicine, University of Minho, Campus Gualtar, 4710-057 Braga, Portugal. Tel: +351 253 604878. Fax: +351 253 604809. Email: [njcsousa@med.uminho.pt](mailto:njcsousa@med.uminho.pt)

**Supplementary Figures**

**
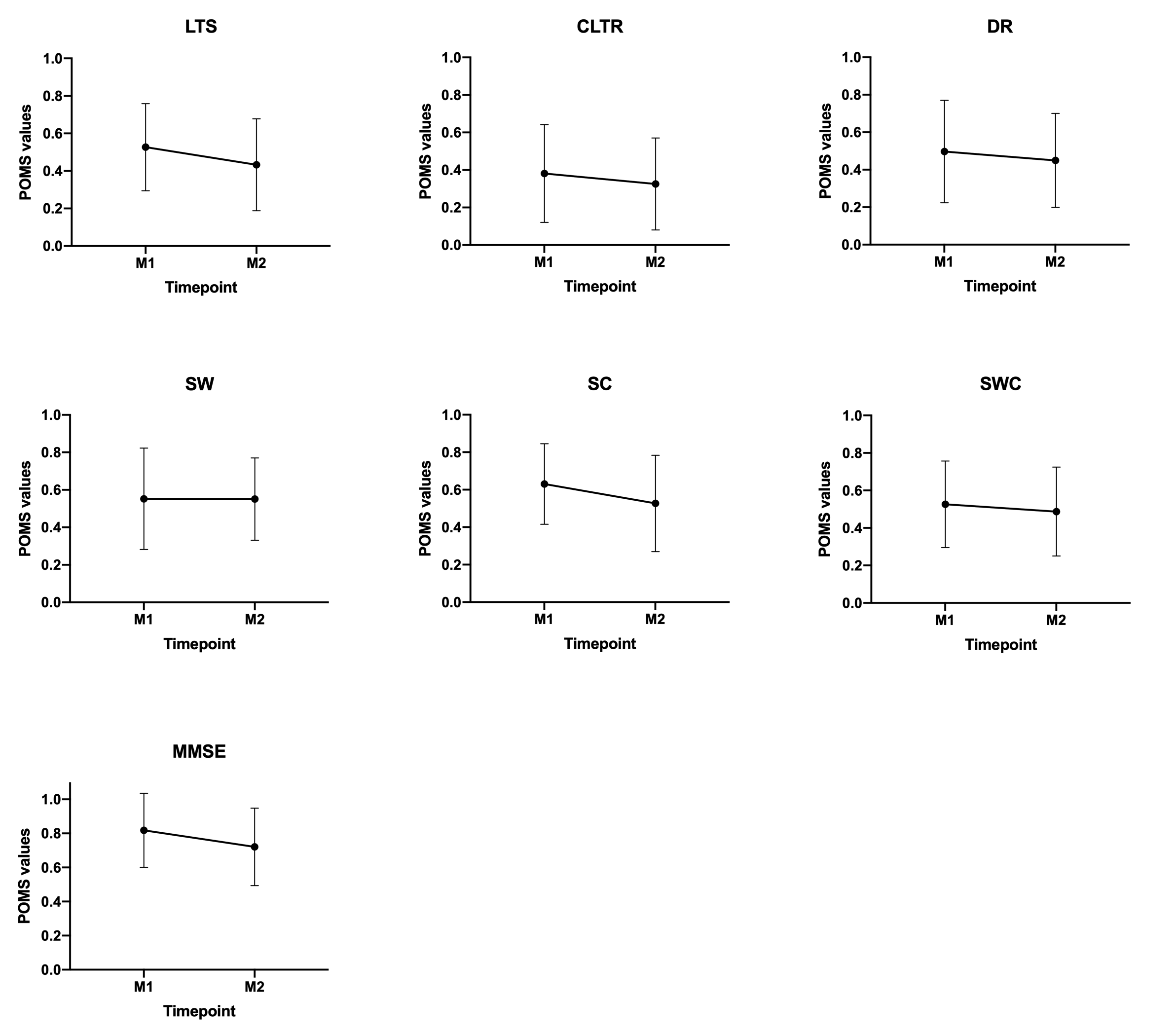
**

**Figure S1 –** Trajectories of cognitive test scores for memory (LTS, CLTR, DR), executive function (SW, SC, SWC) and general cognition (MMSE) along time. The x-axis represents time of assessment and y-axis, the average values of each test. Error bars represent standard deviation. All cognitive test scores exhibit a decrease between timepoints, with the exception of SW that remains practically constant.

**
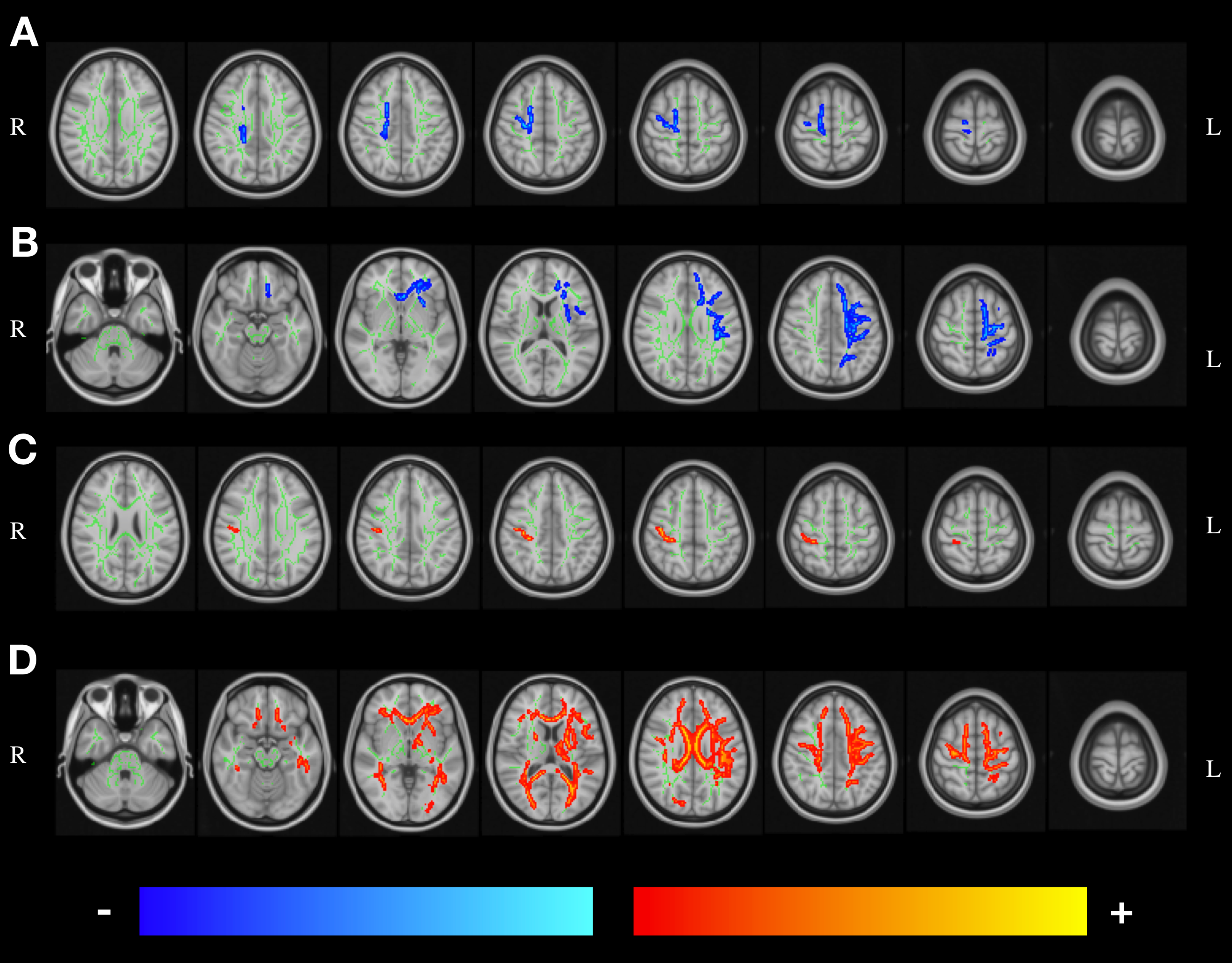
**

**Figure S2 –** Statistically significant changes along time in a) FA Cluster 1, b) FA Cluster 2, c) RD Cluster 1 and d) RD Cluster 2. Blue/light-blue gradient indicates decreases along time. Red/yellow gradient indicate increases along time. All results were considered significant at p<0.05 (FWE corrected for multiple comparisons).

**Supplementary Tables**

| Metric | White Matter Tract | Cluster Size (Number of Voxels) | Peak Coordinates in mm (x, y, z) | Peak Value (1-p) |
| --- | --- | --- | --- | --- |
| FA | Right Posterior corona radiata | 1335 | 19, -29, 35 | 0.984 |
|  | Left Superior corona radiata | 9813 | -18, -23, 36 | 0.999 |
| AD | Unclassified | 28881 | -12, 23, -14 | 1 |
| RD | Unclassified | 533 | 43, -21, 51 | 0.973 |
|  | Unclassified | 35144 | -41, -25, -20 | 1 |
| MD | Unclassified | 35502 | -41, -25, -20 | 1 |

**Table S1** Clusters presenting significant results between timepoints

Abbreviations: FA, fractional anisotropy; AD, axial diffusivity; MD, mean diffusivity; RD, radial diffusivity.

**Table S2 –** Pairwise comparisons between slopes of the different significant clusters of each DTI metric (results Bonferroni corrected at p<0.05).

| Contrast | Test Statistic |
| --- | --- |
| FA Cluster 1 – FA Cluster 2 | t(600) = 0.65, p=1 |
| FA Cluster 1 – AD Cluster 1 | **t(600) = 3.75, p=0.003**** |
| FA Cluster 1 – RD Cluster 1 | **t(600) = 3.75, p=0.003**** |
| FA Cluster 1 – RD Cluster 2 | **t(600) = 3.75, p=0.003**** |
| FA Cluster 1 – MD Cluster 1 | **t(600) = 3.75, p=0.003**** |
| FA Cluster 2 – AD Cluster 1 | **t(600) = 3.10, p=0.03*** |
| FA Cluster 2 – RD Cluster 1 | **t(600) = 3.10, p=0.03*** |
| FA Cluster 2 – RD Cluster 2 | **t(600) = 3.10, p=0.03*** |
| FA Cluster 2 – MD Cluster 1 | **t(600) = 3.10, p=0.03*** |
| AD Cluster 1 – RD Cluster 1 | t(600) = 0.001, p=1 |
| AD Cluster 1 – RD Cluster 2 | t(600) = 0.002, p=1 |
| AD Cluster 1 – MD Cluster 1 | t(600) = 0.002, p=1 |
| RD Cluster 1 – RD Cluster 2 | t(600) = 0.002, p=1 |
| RD Cluster 1 – MD Cluster 1 | t(600) = 0.001, p=1 |
| RD Cluster 2 – MD Cluster 1 | t(600) = -0.0002 , p=1 |

*Abbreviations*: FA, fractional anisotropy; AD, axial diffusivity; MD, mean diffusivity; RD, radial diffusivity.

*Significance codes:* * p < 0.05, ** p < 0.01, *** p < 0.001
